# Comparing AI-Assisted Problem-Solving Ability With Internet Search Engine and e-Books in Medical Students With Variable Prior Subject Knowledge: Cross-Sectional Study

**DOI:** 10.2196/81264

**Published:** 2026-01-06

**Authors:** Ajiith Xavier, Syed Shariq Naeem, Waseem Rizwi, Hiramani Rabha

**Affiliations:** 1 Department of Pharmacology, Jawaharlal Nehru Medical College and Hospital, Aligarh Muslim University, Medical Road, Aligarh, Uttar Pradesh, 202001, India, 91 9634912166

**Keywords:** artificial intelligence, AI, large language models, LLM, medical education, ChatGPT, cognitive performance, subject-naive learners

## Abstract

**Background:**

Artificial intelligence (AI), particularly large language models (LLMs) such as ChatGPT (OpenAI), is rapidly influencing medical education. Its effectiveness for students with varying levels of prior knowledge remains underexplored.

**Objective:**

This study aimed to evaluate the performance of medical students with and without formal pharmacology knowledge when using AI-LLM GPTs, internet search engines, e-books, or self-knowledge to solve multiple-choice questions (MCQs).

**Methods:**

A cross-sectional study was conducted at a tertiary care teaching hospital with 100 medical students, divided into a “naive” group (n=50; no pharmacology training) and a “learned” group (n=50; completed pharmacology training). The study was started after approval from the Institutional Ethics Committee of Jawaharlal Nehru Medical College Hospital, Aligarh Muslim University (1018/IEC/23/8/23). Each participant answered 4 sets of 20 MCQs using self-knowledge, e-books, Google, or ChatGPT-4o. Scores were compared using analysis of covariance with self-knowledge scores as a covariate.

**Results:**

Learned students significantly outperformed naive students across all methods (*P*<.001), with the largest effect size in the AI-LLM GPT set (partial η²=0.328). For both groups, the performance hierarchy was AI-LLM GPT > internet search engine > self-knowledge ≈ e-books. Notably, the naive students who used AI scored higher (mean 13.24, SD 3.31) than the learned students who used Google (mean 12.14, SD 2.01; *P*=.01) or e-books (mean 10.22, SD 3.12; *P*<.001).

**Conclusions:**

AI-LLM GPTs can significantly enhance problem-solving performance in MCQ-based assessments, particularly for students with limited prior knowledge, even allowing them to outperform knowledgeable peers using traditional digital resources. This underscores the potential of AI to transform learning support in medical education, although its impact on deep learning and critical thinking requires further investigation.

## Introduction

Artificial intelligence (AI) is the ability of a digital computer or computer-controlled robot to perform tasks commonly associated with intelligent beings [1]. This includes the ability to reason, discover meaning, generalize, and learn from past experiences [2]. Since the development of digital computers in the 1940s, it has been demonstrated that computers can be programmed to perform extremely complex tasks, such as proving mathematical theorems or playing chess, with great proficiency [3]. AI involves the science and engineering of creating systems capable of performing tasks that require humanlike intelligence, including learning, judgment, and decision-making [4]. AI has successfully solved complex problems in various domains, including education. The application of AI in natural language processing has led to the creation of intelligent chatbots and virtual assistants capable of understanding and producing human language [5].

Popenici and Kerr [6] investigated the impact of AI systems on learning and teaching, highlighting potential conflicts between students and instructors, such as privacy concerns, changes in power structures, and excessive control. These studies have called for further research into the impact of AI systems on learner-instructor interactions to identify gaps, issues, or barriers that prevent AI systems from achieving their intended potential [7].

In the modern era of education, technology has become an integral part of the learning process. The advent of advanced language models such as ChatGPT (OpenAI), along with the vast availability of information on platforms such as Google and e-books, enables educators to enhance students’ educational experiences [7].

Medical education demands not only the acquisition of factual knowledge but also the development of critical thinking and decision-making skills [8]. However, the effectiveness of AI–large language model (LLM) GPT–driven tools in enhancing these competencies remains underexplored, particularly concerning the prior knowledge of learners [9]. Students with strong foundational knowledge can use AI tools to deepen their understanding and sharpen their reasoning. In contrast, those with less prior knowledge may use them to fill gaps in their learning or face some difficulties in comprehension [10].

This study used ChatGPT-4o as the representative AI-LLM GPT tool for 2 reasons. First, ChatGPT was widely accessible to students at the time of study and required minimal technical expertise. Second, it represents the current generation of general-purpose LLMs that students are likely to encounter in real-world educational settings. The most notable advantage is its breadth of training data, which expanded from 175 billion parameters in GPT-3.5 to 1 trillion in the GPT-4.0 model [11]. Bing’s current AI model has 175 billion parameters, and Microsoft’s Bard has 540 billion in comparison [12,13]. The domain-specific LLMs, enriched with pharmacology databases or specialized medical AI agents, may offer better performance characteristics, but widespread use of such tools is still limited due to paywall and validation issues.

The aim of this study is to compare the effectiveness of AI-LLM GPT tools (ChatGPT-4o), internet search engines (Google), e-books, and self-knowledge for answering pharmacology multiple-choice questions (MCQs) among medical students with different levels of prior pharmacology knowledge (naive vs learned groups). The study explores the assistance provided by AI-LLM GPT tools in the ability of problem solving using a cross-sectional design.

## Methods

### Ethical Considerations

This was a cross-sectional study conducted at a tertiary care teaching hospital. The study was started after approval from the Institutional Ethics Committee of Jawaharlal Nehru Medical College Hospital, Aligarh Muslim University (1018/IEC/23/8/23). All participants were adult MBBS students and provided written informed consent after receiving information about the study purpose and procedures. Data were collected anonymously using coded identifiers. No personal or identifiable information was recorded or published. Participants received no monetary or academic compensation.

### Setting and Participants

Participants included 50 second-year medical students who had just joined the second year and 50 third-year students who had recently passed their second year (ie, N=100). The second-year students, designated as the “naive group,” had no prior formal exposure to pharmacology coursework. The third-year students, designated as the “learned group,” had completed a comprehensive 1-year pharmacology curriculum during their second year. This curriculum consisted of 80 hours of didactic lectures, 150 hours of interactive teaching, including practical laboratory sessions, case-based learning modules, self-directed learning modules, short group teaching, and regular assessments, including sessional and professional examinations. Students had completed this coursework approximately 2‐3 months prior to participation in this study. The curriculum covered topics such as general pharmacology, autonomic pharmacology, cardiovascular drugs, and antimicrobials, aligned with the medical council syllabus for undergraduate medical education. All learned group participants had passed their second-year pharmacology examination with a minimum score of 50%.

### Study Development

Prior to the assessment, all participants received a 15-minute standardized orientation session on using ChatGPT and were provided with an eBook of Pharmacology. This training covered basic prompt formulation and how to ask follow-up questions. Students accessed ChatGPT through computer workstations in a controlled examination hall environment to ensure standardized conditions. For the Google search set, students used desktop computers with standard Google Chrome or Mozilla Firefox browsers. For the e-book set, students were provided with PDF versions of a pharmacology textbook accessible on the same computers. The training session was conducted using a standardized presentation to maintain homogeneity of assistance tool exposure. The self-knowledge set required no external tools. All sessions were proctored to ensure compliance with assigned methods for each question set.

The 80 MCQs were divided into four parallel sets (A, B, C, and D), each containing 20 questions. To ensure equivalence across sets, questions were matched for content area coverage (eg, autonomic, cardiovascular, and chemotherapy) [1], question type (theory, calculation, or image-based) [2], and estimated difficulty level based on faculty assessment [3].

**Figure 1.**
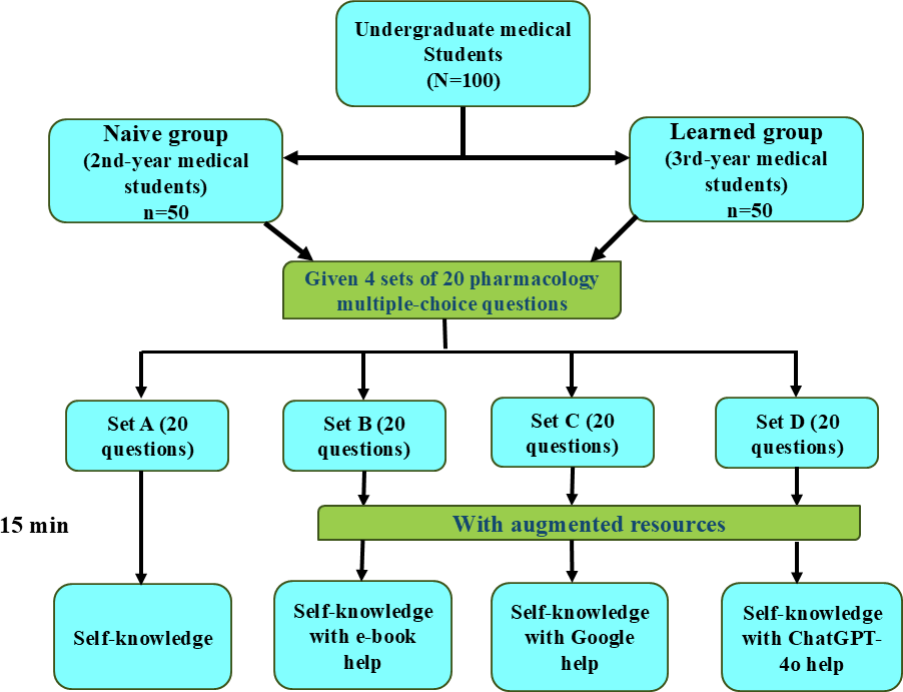
Methodology flowchart.

Students had 15 minutes to respond to each set of questions. They were instructed to respond to the questions in a manner that self-knowledge was used to answer questions in set A; e-books were used to answer questions in set B; internet search engine (Google) was used to answer questions in set C; and AI-LLM GPT (ChatGPT) was used to answer questions in set D. Each correct answer was given 1 mark, and no negative marking was given. The methodology flowchart of this study is shown in Figure 1.

### Statistical Analysis

Data analysis was done using SPSS (version 23; IBM Corp) and R software package (R Foundation for Statistical Computing). The mean (the average number of correctly answered questions out of the 20 MCQs included in each augmentation set such as self-knowledge, e-book, internet search engine, and GPT-4o) between different groups was assessed using the independent test. Intergroup and intragroup comparisons were conducted using analysis of covariance (ANCOVA), with self-knowledge group (set A) being a covariate. A *P* value <.05 was considered to indicate statistical significance.

## Results

The intergroup comparison between the learned and naive student groups was analyzed using ANCOVA. The estimated marginal means, partial η² values, and statistical significance for each group are presented in Table 1. The self-knowledge set was used as a covariate in the analysis to control for baseline differences in knowledge across the sets. This approach allowed for a more accurate assessment of the effects of the augmented resources methods (ChatGPT-4o, Google, and e-books) on student performance.

**Table 1.** Intergroup comparison between the learned and naive using analysis of covariance (N=100).

Augmentation resource and group		Score, mean (SD)	Estimated marginal mean	Partial η²	*P* value
AI^a^-LLM^b^ GPT (ChatGPT-4o)				0.328	<.001
	Naive	13.24 (3.31)	13.18		
	Learned	15.42 (1.89)	15.48		
Internet search engine (Google)				0.241	<.001
	Naive	10.82 (2.13)	10.76		
	Learned	12.14 (2.01)	12.20		
e-Books				0.195	<.001
	Naive	5.82 (2.77)	5.78		
	Learned	10.22 (3.12)	10.26		

a AI: artificial intelligence.

b LLM: large language model.

The analysis of estimated marginal means revealed that the learned students consistently outperformed the naive students across all sets. In the AI-LLM GPT (ChatGPT-4o) set, the learned students achieved an estimated marginal mean of 15.48 compared to 13.18 for the naive students, with a large effect size (partial η²=0.328, *P*<.001). In the internet search engine (Google) set, the estimated marginal mean for the learned students was 12.20, whereas the naive students scored 10.76, with a moderate effect size (partial η²=0.241; *P*<.001). The learned students in the e-book set had an estimated marginal mean of 10.26, whereas the naive students had 5.78, a moderate effect size (partial n^2^=0.195; *P*<.001).

The post hoc analysis for the naive group shows that the AI-LLM GPT-assisted method resulted in the highest performance when compared to other methods like internet search engine–based learning, self-knowledge, and e-books methods. A significant difference was found in all pairwise comparisons (*P*<.001) except for e-books versus self-knowledge, which was not statistically significant (mean difference=−0.30; *P*=.91). The performance hierarchy observed was AI-LLM GPT versus self-knowledge (*P*<.001) **>** internet search engine versus self-knowledge (*P*<.001) **>** self-knowledge ≈ e-books (*P*=.91), showing the superior effectiveness of AI tools for naive learners.

The post hoc analysis for the learned group shows that the AI-LLM GPT–assisted learning method resulted in the highest performance when compared to other methods, whereas self-knowledge and e-books methods showed the lowest and statistically similar outcomes (mean difference=−0.72, *P*=.33). Significant differences were observed between Google and self-knowledge sets (*P*=.04), ChatGPT-4o and self-knowledge sets (*P*<.001), Google and e-books sets (*P*<.001), ChatGPT-4o and e-books (*P*<.001), and ChatGPT-4o and Google (*P*<.001), indicating a superior performance across these comparisons. The overall performance hierarchy observed was: AI-LLM GPT > internet search engine > self-knowledge ≈ e-books, indicating the superior effectiveness of AI-LLM GPT in enhancing learning outcomes, even for participants with prior knowledge (Multimedia Appendix 1).

**Figure 2.**
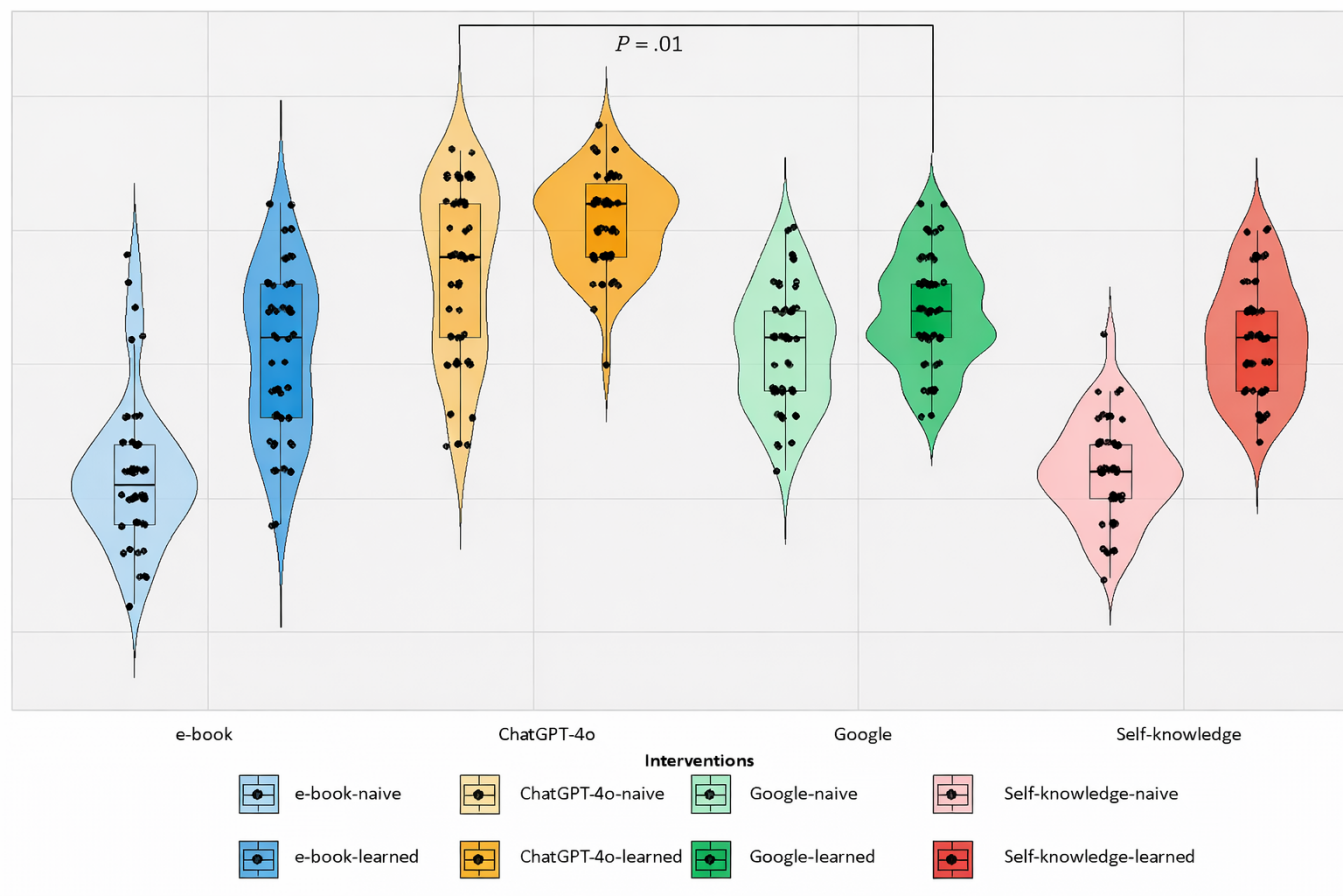
Box-violin plot showing the mean scores in the naive and learned groups.

The mean comparison between the sets, as presented in Figure 2, highlights that learned students consistently outperformed naive students across all categories, as expected. The AI-LLM set achieved the highest performance, with a mean score of 15.3 (SD 1.776). Conversely, the lowest mean was observed in the e-books set, in which learned students scored 10.3 (SD 3.1) and naive students scored 5.7 (SD 2.7).

Interestingly, the self-knowledge sets performed better than the e-books sets, with naive group students achieving a mean score of 6.0 (SD 1.9) and learned group students scoring a mean of 10.96 (SD 2.2). This may be due to the fact that students in the e-book sets spent sufficient time searching for relevant chapters instead of focusing on answering the questions. This led to some questions being left unanswered that likely contributed to their lower overall performance.

While ANCOVA analysis confirmed that the learned students consistently outperformed the naive students when comparing the same learning method (as shown in Table 1), an important cross-method comparison emerged: the naive students who used ChatGPT-4o achieved higher scores (mean 13.2, SD 3.3) than the learned students who used self-knowledge (mean 10.96, SD 2.5), e-books (mean 10.22, SD 3.12), or Google (mean 12.14, SD 2.01). This demonstrates that the advantage conferred by using ChatGPT-4o was sufficient to overcome the knowledge gap between the naive and learned students when the latter were restricted to conventional resources.

## Discussion

### Principal Findings

AI is changing the face of medical education, as it suggests some new solutions to the old forms of teaching, increases the educational experience, and improves the results of students [14,15]. AI-based solutions, including LLMs, such as ChatGPT, virtual assistants, and AI-based simulation technologies, can change how medical students learn and apply their knowledge [16,17].

This study found that learned students consistently outperformed naive students across all learning methods. However, the naive students who used ChatGPT-4o achieved higher scores than the learned students who used conventional resources (ie, self-knowledge, e-books, or Google), suggesting that AI-LLM tools may help bridge knowledge gaps more effectively than traditional learning resources.

Our study demonstrates that AI-powered LLMs such as ChatGPT can notably improve students’ performance on pharmacology MCQs, particularly for those without prior subject knowledge. Although our findings show that students achieved higher MCQ test scores when using ChatGPT-4o, we cannot conclude that this represents a genuine improvement in cognitive performance, critical thinking, or clinical decision-making skills. Higher scores may reflect the AI’s ability to rapidly retrieve and present relevant information rather than deeper student understanding. The findings indicated that learned students have outperformed naive students in all 4 sets, and AI-LLM GPT recorded the best performance. The greatest effect size was observed in the AI group (partial η²=0.328), which indicates the tremendous influence of AI-LLM in ensuring the students improve their understanding and problem-solving skills. These results are consistent with those of previous research in that AI tools may enhance critical thinking and support learning under the condition that they are properly used by a student who already has a good knowledge grounding [18].

As opposed to other methods, the e-books set exhibited the lowest performance. This can be explained by the fact that it takes a lot of time to navigate and search relevant information in the e-books. This reveals the difficulties of self-learning with the help of conventional digital sources, especially in the case of naive students who are probably not able to manage their time efficiently. In contrast, the self-knowledge set outperformed the e-books set, which means that dependence on inherent knowledge, although restricted to a certain extent, can be more helpful than the effective utilization of external means.

Although our quantitative data demonstrate that ChatGPT-4o enhanced student performance, particularly for naive learners, the mechanisms underlying this effectiveness warrant deeper exploration. There may be several reasons for AI-LLM GPT (ChatGPT-4o) to outperform other methods of assistance.

First, ChatGPT-4o provides immediate, targeted responses without requiring students to navigate complex search results or textbook indices. Informal feedback from participants suggested that students who used Google often struggled to identify authoritative sources among numerous search results, whereas those who used e-books spent considerable time locating relevant chapters. In contrast, ChatGPT-4o delivered direct answers to questions, reducing the cognitive load related to information seeking.

Second, ChatGPT-4o’s conversational interface may facilitate iterative learning. Students could ask follow-up questions to clarify concepts, request examples, or seek a specific explanation for a specific MCQ—a dynamic interaction not possible with static e-books and more efficient than reformulating multiple Google searches.

Third, the structured, synthesized format of ChatGPT-4o responses may be particularly beneficial for naive learners who lack the prior knowledge framework to evaluate and integrate fragmented information from multiple sources. ChatGPT-4o essentially predigests information, whereas Google and e-books require students to perform this synthesis themselves—a task that may be especially challenging without foundational knowledge.

However, these remain as hypotheses. Future research should use mixed methods approaches, including think-aloud protocols during tool use, posttask interviews exploring student decision-making processes, and screen recordings analyzing search strategies and information evaluation patterns. Such qualitative data would provide richer insights into how students with different knowledge levels interact with various resources and why certain tools prove more effective. The study of recognizing the pattern of wrong responses in the AI-LLM GPTs output may also generate an “array of errors” for further training and improving LLMs.

These findings have substantial implications for both local and global medical education practices. In our institutional context, where students face high patient loads and limited access to senior clinicians for immediate consultation, AI-LLM GPTs could serve as readily available reference tools to support clinical decision-making during training. However, integration must be carefully structured to enhance rather than replace foundational learning.

Globally, these results are particularly relevant for resource-limited settings where access to comprehensive textbooks, updated references, and expert faculty may be constrained. AI-LLM GPTs could help democratize access to medical knowledge, potentially reducing disparities between well-resourced and under-resourced educational institutions. However, this assumes reliable internet connectivity and technological infrastructure, which remain barriers in many settings.

From a pedagogical perspective, our findings suggest a paradigm shift may be necessary in how we structure medical curricula. Rather than focusing exclusively on memorization of facts—information that AI can rapidly retrieve—educational programs should prioritize teaching students how to (1) formulate effective questions and search strategies, (2) critically evaluate AI-generated responses for accuracy and clinical appropriateness, (3) integrate AI-provided information with clinical context and patient-specific factors, and (4) develop metacognitive skills to recognize the limitations of both their own knowledge and AI tools.

Importantly, although AI-LLMs demonstrated effectiveness for MCQ performance, medical practice requires competencies beyond factual knowledge, including physical examination skills, procedural expertise, empathic patient communication, ethical reasoning under uncertainty, and team-based care coordination. Educational programs must ensure that AI integration enhances rather than diminishes these essential human dimensions of medical practice.

An important limitation is our focus on general-purpose ChatGPT-4o rather than specialized medical or pharmacology-specific AI agents. Domain-enriched LLMs that integrate pharmacology textbooks or medical databases may demonstrate different performance characteristics and potentially offer more accurate, context-specific responses. However, our choice reflects the current reality that most students have access to general LLMs such as ChatGPT-4o rather than specialized medical AI tools and makes the study more generalizable than using domain-enhanced LLMs. Second, the cross-sectional, single-day design prevents assessment of long-term learning outcomes, knowledge retention, or the development of clinical reasoning skills. Similarly, the sequential exposure to different resources (sets A through D) may have introduced order effects or learning transfer between sets. To minimize the learning transfer, the questions have been chosen from diverse topics and moderated to remove any repetition. However, the order effect cannot be removed completely in these types of cross-sectional–sequential studies.

AI will be able to deliver personalized learning and adjust to the needs of students. This individualization helps the learners make their progress through their priorities, repeating difficult concepts or working through material as they become familiar with each subject [19,20]. The AI-powered platforms can monitor student performance in real-time and can track the areas where the students might need more education or practice [21]. Such benefits help achieve the speed and the depth of knowledge acquisition with a higher level of efficiency and more targeted learning [22,23].

### Conclusions

To conclude, this study shows that the learned group outperformed the naive group in all sets, including AI-LLM GPT, internet search engine, e-books, and self-knowledge in problem solving. Ironically, the AI-LLM GPT naive group outperformed the problem-solving skills of even the learned group augmented with an internet search engine, showcasing the disruptive potential of AI-LLM in medical education. Further longitudinal studies examining knowledge retention, clinical reasoning development, and the ability to solve novel problems without AI assistance are needed to determine whether AI tools genuinely enhance cognitive capabilities or primarily serve as effective reference tools.

## Supplementary material

10.2196/81264 Multimedia Appendix 1 Post hoc results of the naive and learned groups.

## Abbreviations

AI: artificial intelligence
ANCOVA: analysis of covariance
LLM: large language model
MCQ: multiple-choice question
