# Supplementary material for: Comparing AI-Assisted Problem-Solving Ability With Internet Search Engine and e-Books in Medical Students With Variable Prior Subject Knowledge: Cross-Sectional Study
Source: JMIR Med Educ. 2026 Jan 6;12:e81264. doi: 10.2196/81264 (PMC12772426; doi:10.2196/81264)
Supplement: Multimedia Appendix 1 [file mededu-v12-e81264-s001.docx]

**Supplementary Table S1** Retrieval strategy

| Database | Platform / Provider | Date of Search | Search number | Retrieval strategy | Limits / Filters Applied at Search | Results |
| --- | --- | --- | --- | --- | --- | --- |
| PubMed | NCBI | 2025-09-24 | #1 | ((((((Non-alcoholic Fatty Liver Disease) OR (metabolic associated fatty liver disease)) OR (Fatty Liver)) OR (Liver Diseases)) OR (Steatohepatitis)) OR (MAFLD)) OR (NAFLD) | None | 1228 |
|  |  |  | #2 | ((((((machine learning) OR (Deep learning)) OR (artificial intelligence)) OR (artificial neural network)) OR (External validation)) OR (Convolutional Neural Network)) OR (CNN) |  |  |
|  |  |  | #3 | (Hepatic Steatosis) OR (fatty degeneration) |  |  |
|  |  |  | #1 AND #2 AND #3 | |  |  |
| Cochrane library | Wiley | 2025-09-24 | #1 | (Non-alcoholic Fatty Liver Disease):ti,ab,kw OR (metabolic associated fatty liver disease):ti,ab,kw OR (Fatty Liver):ti,ab,kw OR (Liver Diseases):ti,ab,kw OR (Steatohepatitis):ti,ab,kw | None | 31 |
|  |  |  | #2 | (machine learning):ti,ab,kw OR (Deep learning):ti,ab,kw OR (Convolutional Neural Network):ti,ab,kw OR (artificial intelligence):ti,ab,kw OR (External validation):ti,ab,kw |  |  |
|  |  |  | #3 | (Hepatic Steatosis):ti,ab,kw OR (fatty degeneration):ti,ab,kw OR (Steatosis of Liver):ti,ab,kw OR (Visceral Steatosis):ti,ab,kw OR (Liver Steatosis):ti,ab,kw |  |  |
|  |  |  | #1 AND #2 AND #3 | |  |  |
| Embase | Elsevier | 2025-09-24 | #1 | 'hepatic steatosis':ti,ab,kw OR 'fatty degeneration':ti,ab,kw OR 'steatosis of liver':ti,ab,kw OR 'visceral steatosis':ti,ab,kw OR 'liver steatosis':ti,ab,kw | None | 421 |
|  |  |  | #2 | 'machine learning':ti,ab,kw OR 'deep learning':ti,ab,kw OR 'artificial intelligence':ti,ab,kw OR 'artificial neural network':ti,ab,kw OR 'external validation':ti,ab,kw OR 'convolutional neural network':ti,ab,kw OR cnn:ti,ab,kw |  |  |
|  |  |  | #3 | 'non-alcoholic fatty liver disease':ti,ab,kw OR 'metabolic associated fatty liver disease':ti,ab,kw OR 'fatty liver':ti,ab,kw OR 'liver diseases':ti,ab,kw OR steatohepatitis:ti,ab,kw OR nafld:ti,ab,kw OR mafld:ti,ab,kw |  |  |
|  |  |  | #1 AND #2 AND #3 | |  |  |
| Web of Science | Clarivate | 2025-09-24 | #1 | ((((((TS=(machine learning)) OR TS=(Deep learning)) OR TS=(artificial intelligence)) OR TS=(artificial neural network)) OR TS=(External validation)) OR TS=(Convolutional Neural Network)) OR TS=(CNN) and Preprint Citation Index (Exclude – Database) | Source: Preprint Citation Index was excluded. | 712 |
|  |  |  | #2 | ((((TS=(Hepatic Steatosis)) OR TS=(fatty degeneration)) OR TS=(Steatosis of Liver)) OR TS=(Visceral Steatosis)) OR TS=(Liver Steatosis) and Preprint Citation Index (Exclude – Database) |  |  |
|  |  |  | #3 | ((((((TS=(Non-alcoholic Fatty Liver Disease)) OR TS=(metabolic associated fatty liver disease)) OR TS=(Fatty Liver)) OR TS=(Liver Diseases)) OR TS=(Steatohepatitis)) OR TS=(NAFLD)) OR TS=(MAFLD) and Preprint Citation Index (Exclude – Database) |  |  |
|  |  |  | #1 AND #2 AND #3 and Preprint Citation Index (Exclude – Database) | |  |  |
| IEEE Xplore | IEEE | 2025-09-24 | #1 | ((Non-alcoholic Fatty Liver Disease) OR (metabolic associated fatty liver disease) OR (Fatty Liver) OR (Liver Diseases) OR (Steatohepatitis) OR (NAFLD) OR (MAFLD)) AND ((Hepatic Steatosis) OR (fatty degeneration) OR (Steatosis of Liver) OR (Visceral Steatosis) OR (Liver Steatosis)) | None | 144 |
| Other supplementary searches | No additional sources (e.g., grey literature, reference list checking) were searched beyond the electronic databases listed above. | | | | | |

**
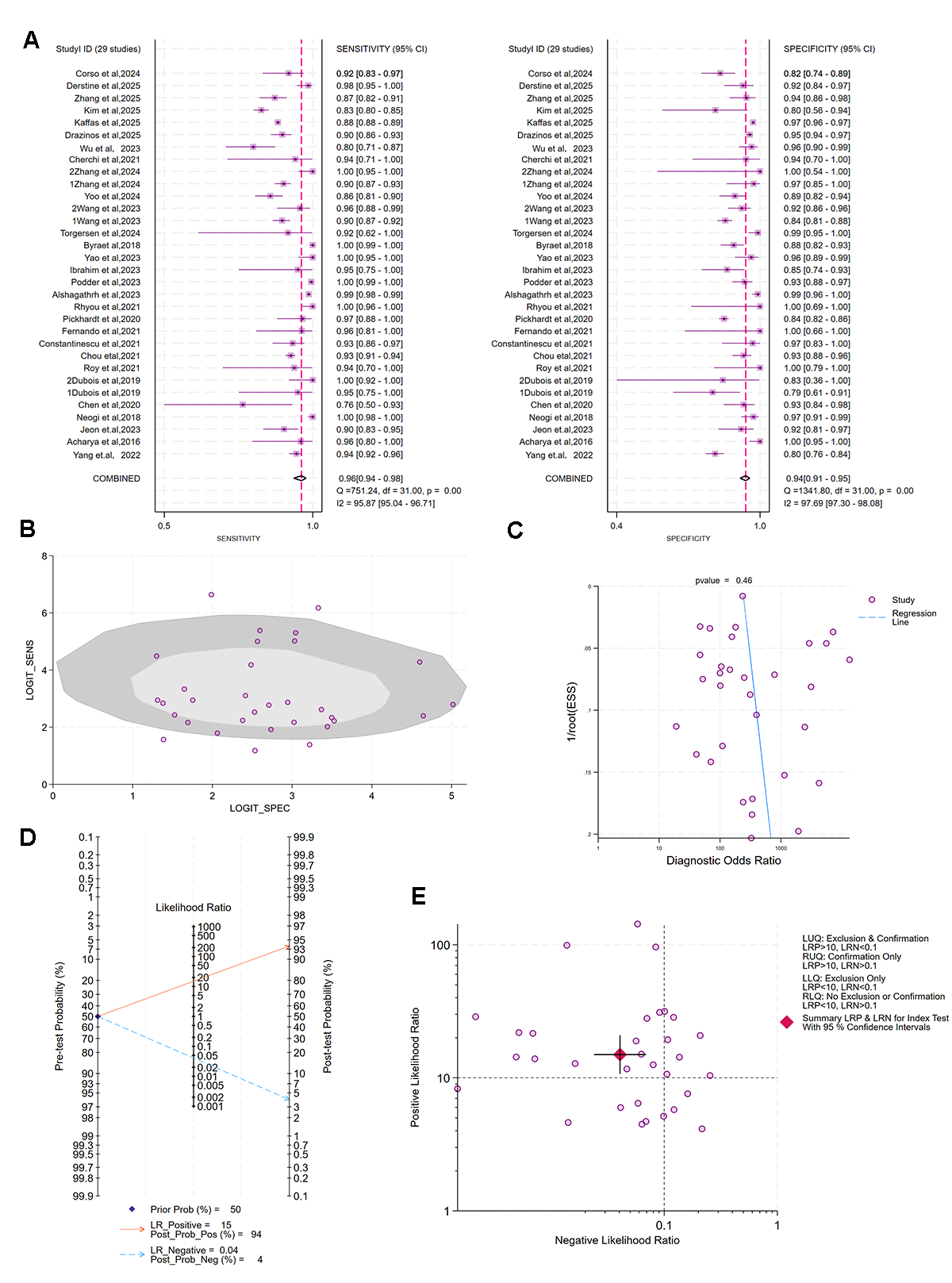
Supplementary Figure S1. AI Types - DL**

A) Forest plots of sensitivity and specificity for the DL subgroup (29 studies, 32 datasets); B) bivariate boxplot illustrating distribution and heterogeneity; C) Deeks’ funnel plot assessing potential publication bias; D) Fagan’s nomogram depicting post-test probability; E) clinical application plot of LRP and LRN.

**
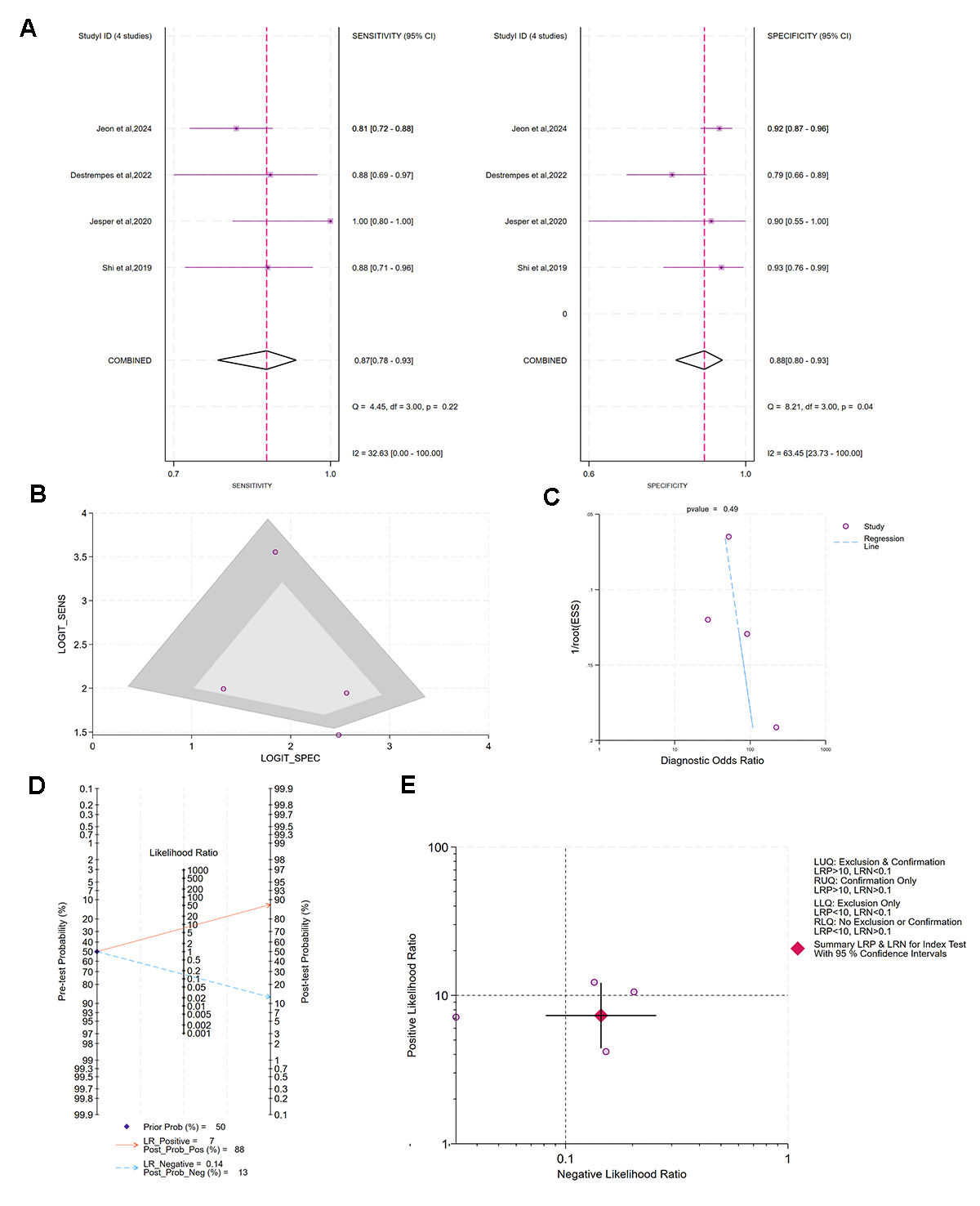
Supplementary Figure S2. AI Types - ML**

A) Forest plots of sensitivity and specificity for the ML subgroup (4 studies, 4 datasets). B) Bivariate boxplot illustrating distribution and heterogeneity. C) Deeks’ funnel plot assessing potential publication bias. D) Fagan’s nomogram depicting post-test probability. E) Clinical application plot of LRP and LRN.

**
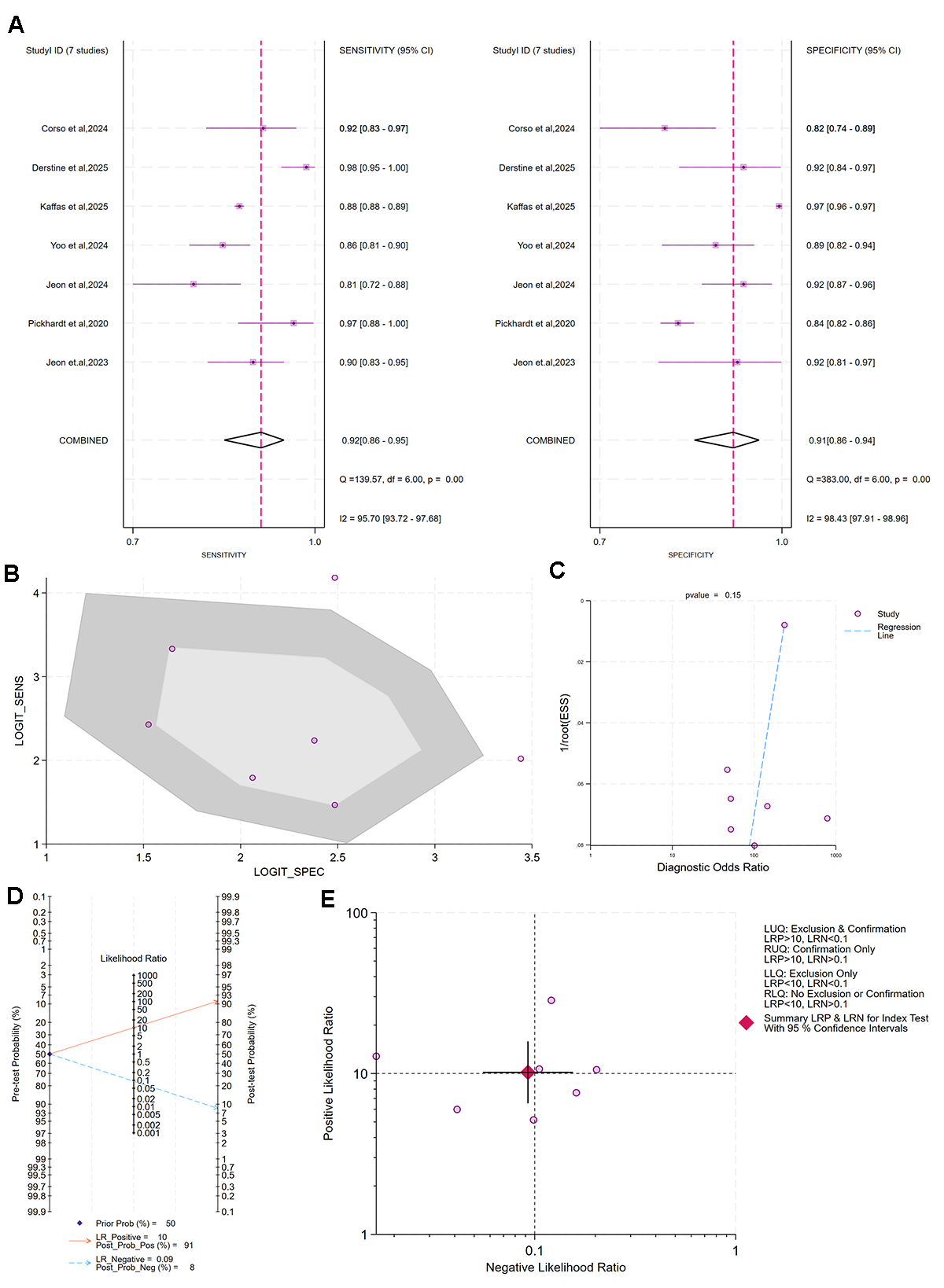
Supplementary Figure S3. Reference Standard - MRI-PDFF**

A) Forest plots of sensitivity and specificity for the MRI-PDFF subgroup (7 studies, 7 datasets). B) Bivariate boxplot illustrating distribution and heterogeneity. C) Deeks’ funnel plot assessing potential publication bias. D) Fagan’s nomogram depicting post-test probability. E) Clinical application plot of LRP and LRN.

**
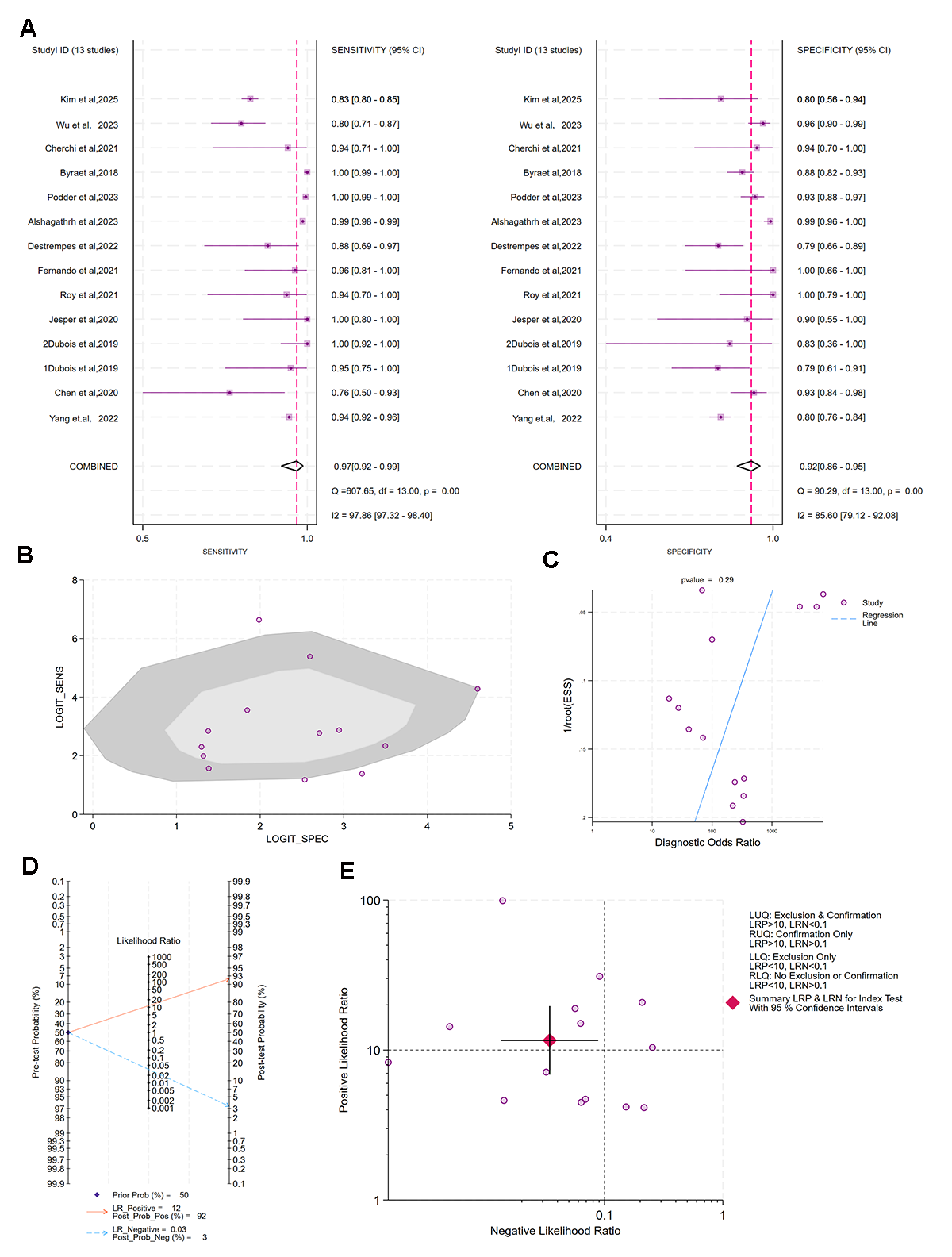
Supplementary Figure S4. Reference Standard - Pathology**

A) Forest plots of sensitivity and specificity for the pathology subgroup (13 studies, 14 datasets). B) Bivariate boxplot illustrating distribution and heterogeneity. C) Deeks’ funnel plot assessing potential publication bias. D) Fagan’s nomogram depicting post-test probability. E) Clinical application plot of LRP and LRN.

**
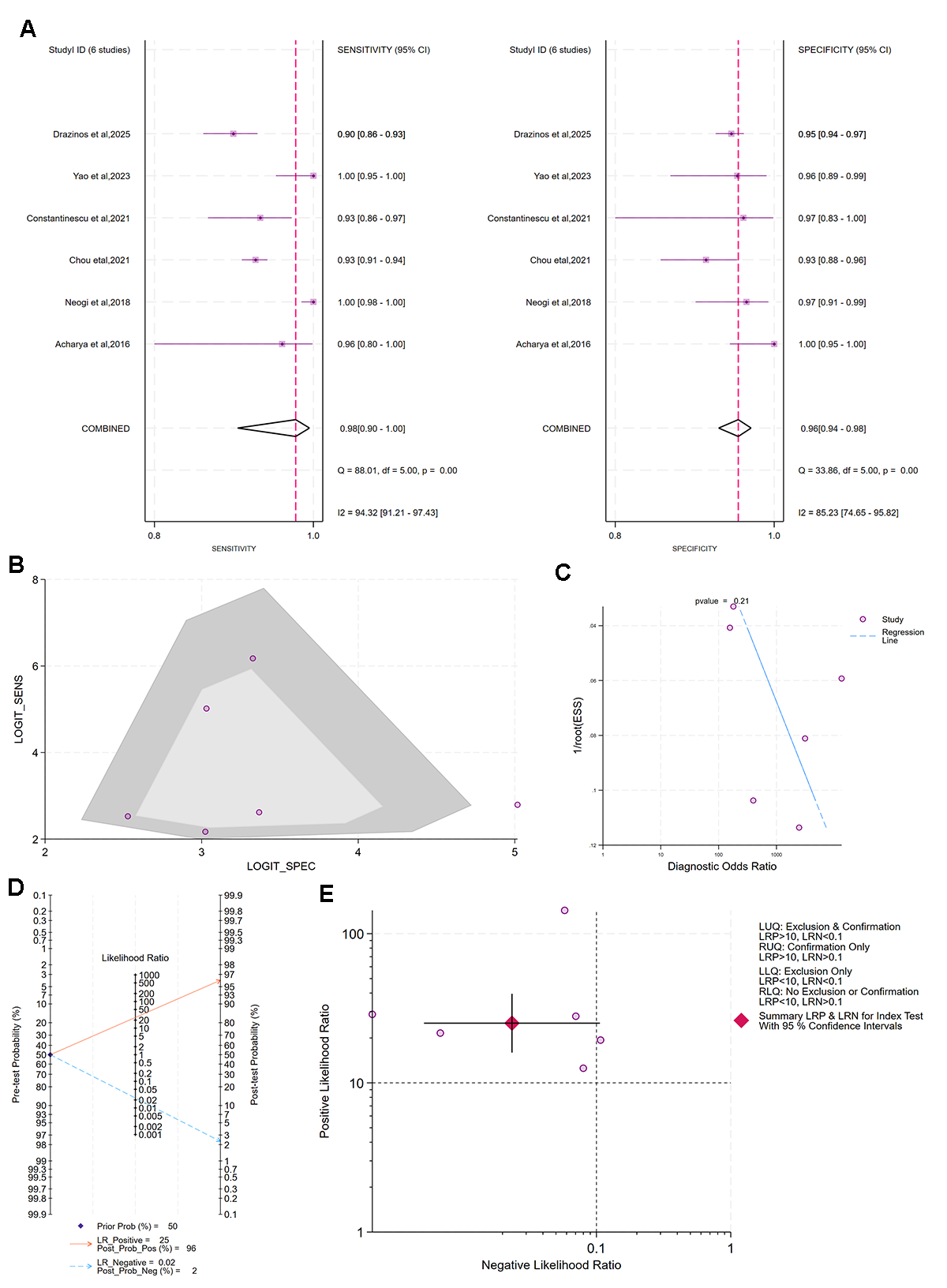
Supplementary Figure S5. Reference Standard - US**

A) Forest plots of sensitivity and specificity for the US subgroup (6 studies, 6 datasets). B) Bivariate boxplot illustrating distribution and heterogeneity. C) Deeks’ funnel plot assessing potential publication bias. D) Fagan’s nomogram depicting post-test probability. E) Clinical application plot of LRP and LRN.

**
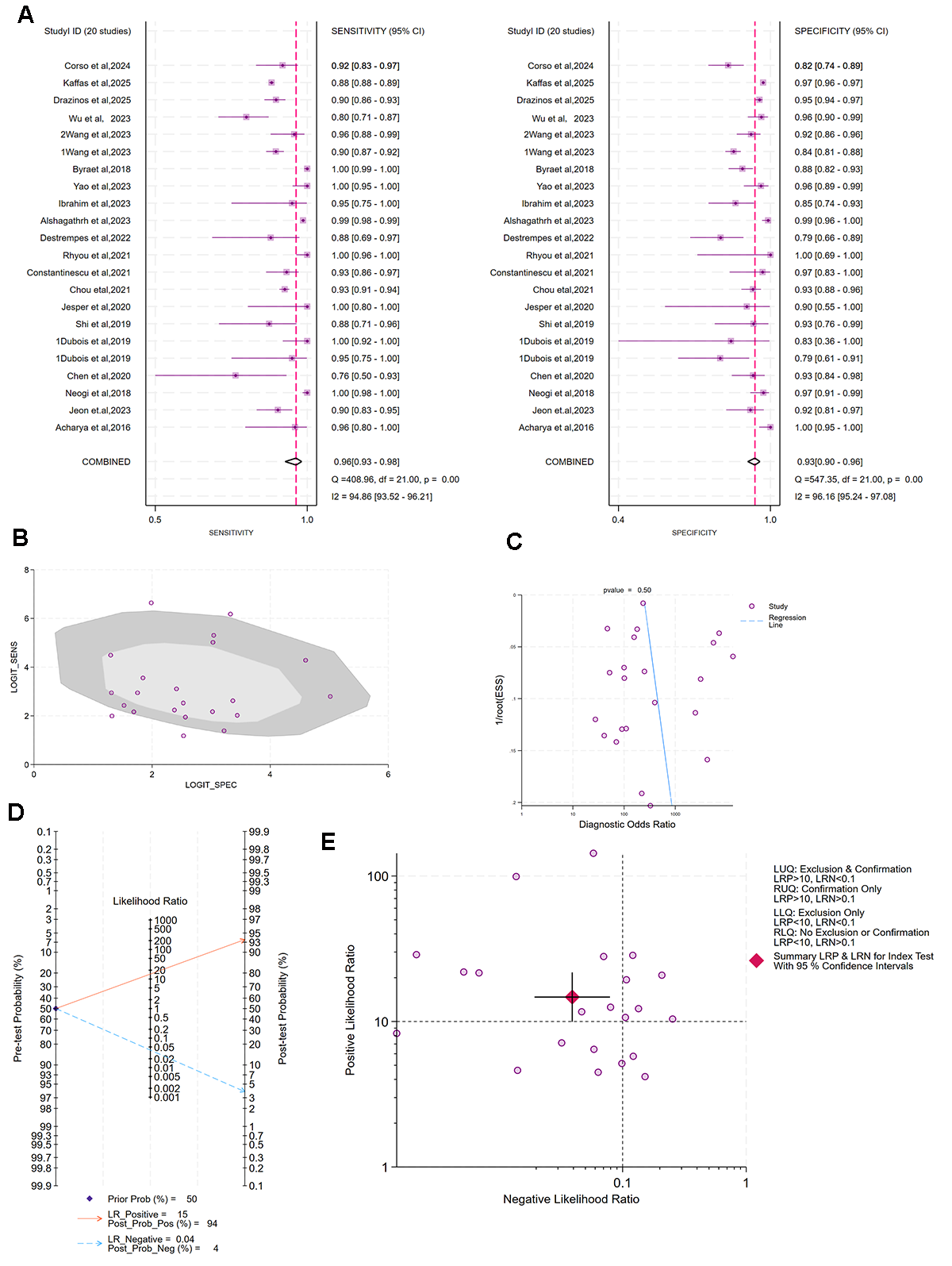
Supplementary Figure S6. Imaging Modalities - US**

A) Forest plots of sensitivity and specificity for US imaging (20 studies, 22 datasets). B) Bivariate boxplot illustrating distribution and heterogeneity. C) Deeks’ funnel plot assessing potential publication bias. D) Fagan’s nomogram depicting post-test probability. E) Clinical application plot of LRP and LRN.

**
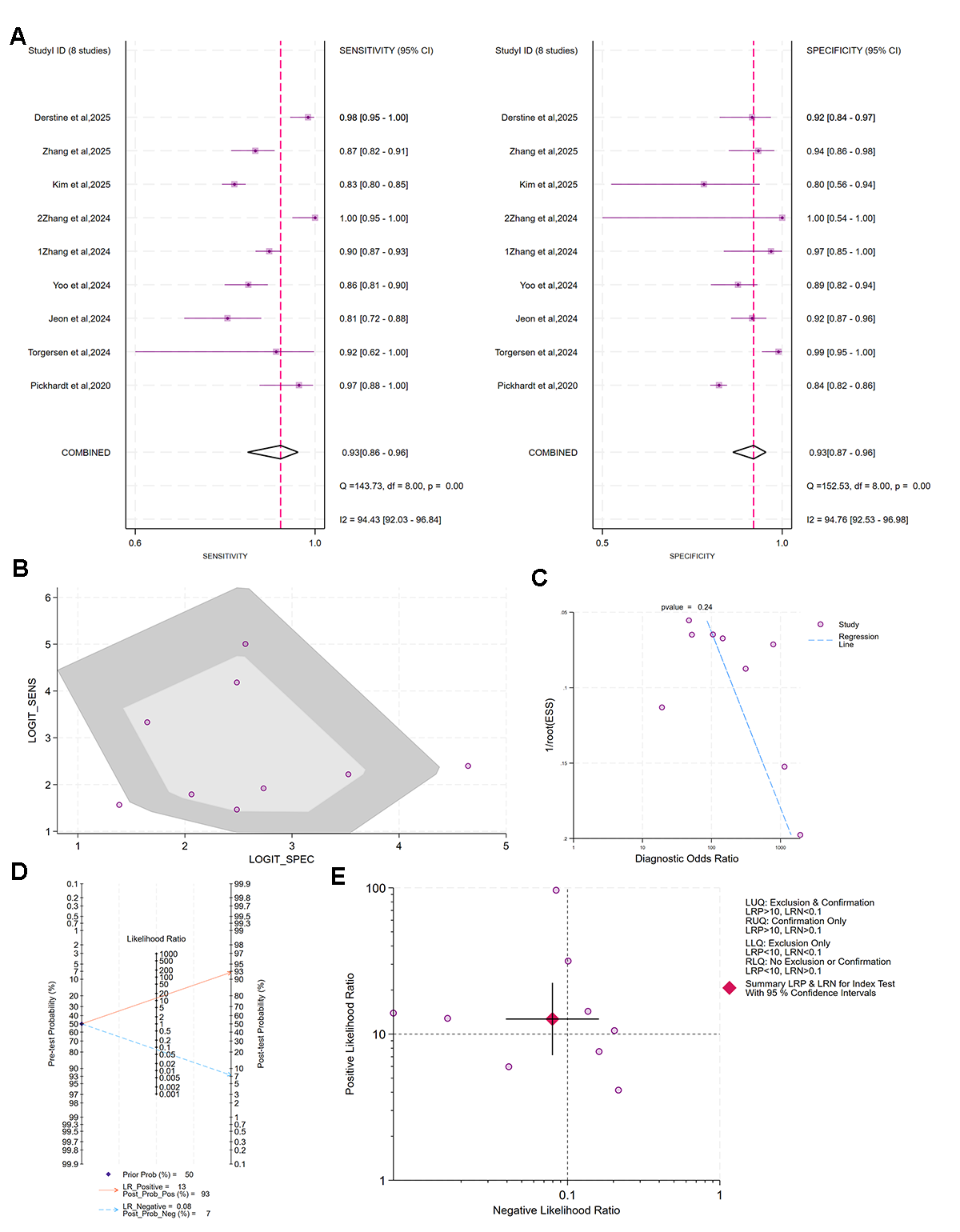
Supplementary Figure S7. Imaging Modalities - CT**

A) Forest plots of sensitivity and specificity for CT imaging (8 studies, 9 datasets). B) Bivariate boxplot illustrating distribution and heterogeneity. C) Deeks’ funnel plot assessing potential publication bias. D) Fagan’s nomogram depicting post-test probability. E) Clinical application plot of LRP and LRN.

**
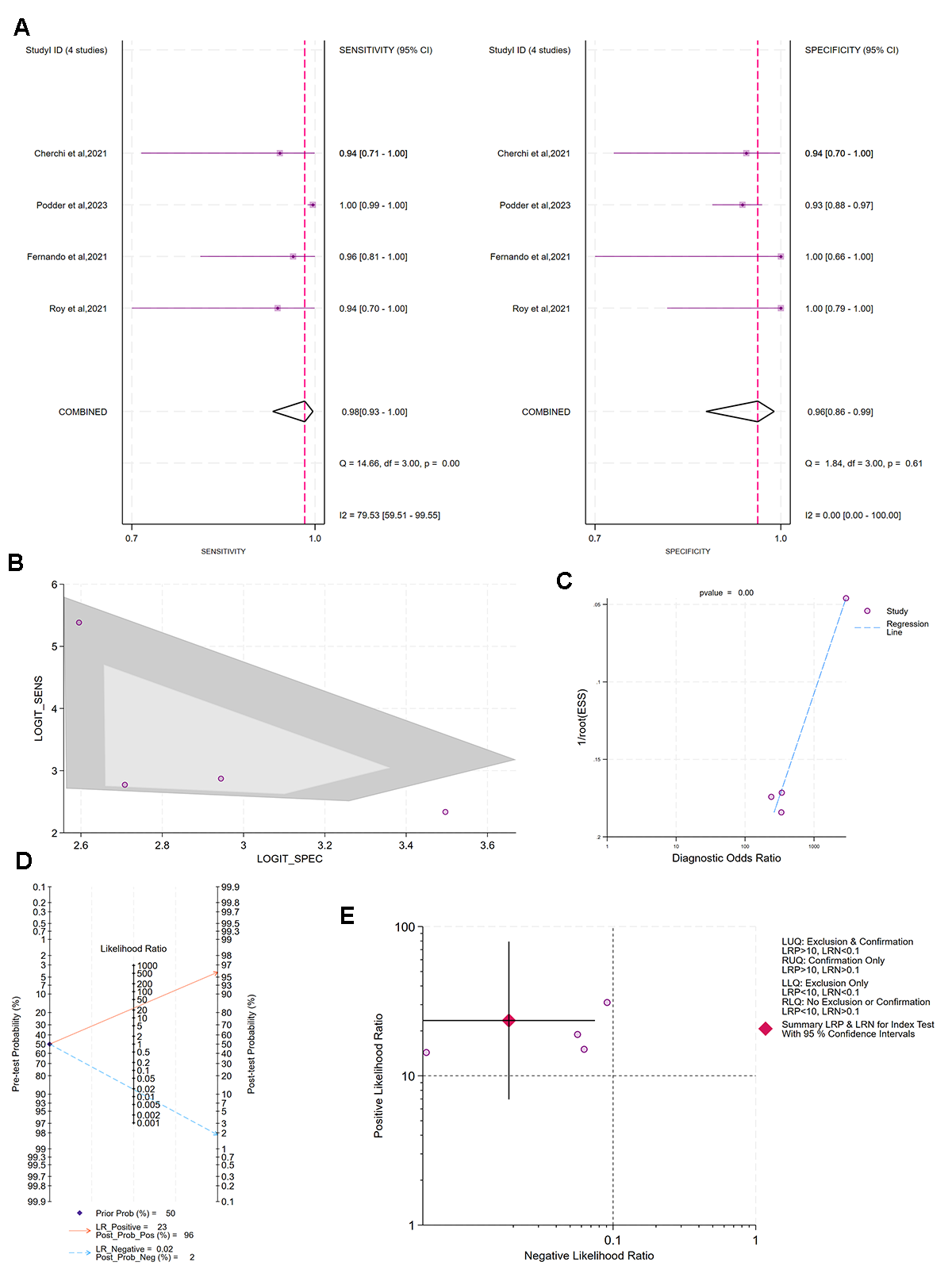
Supplementary Figure S8. Imaging Modalities - Pathology**

A) Forest plots of sensitivity and specificity for pathology imaging (4 studies, 4 datasets). B) Bivariate boxplot illustrating distribution and heterogeneity. C) Deeks’ funnel plot assessing potential publication bias. D) Fagan’s nomogram depicting post-test probability. E) Clinical application plot of LRP and LRN.

**
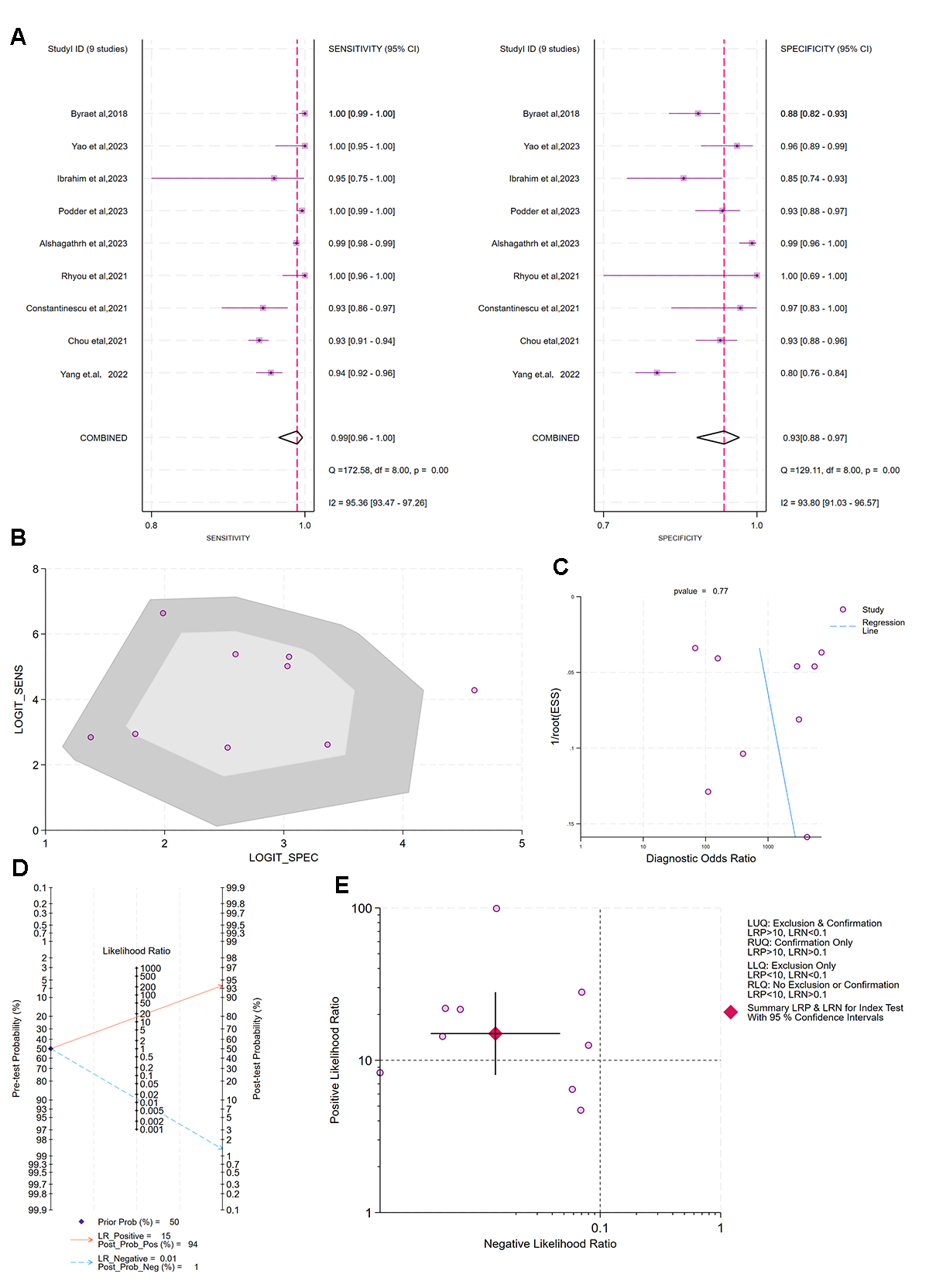
Supplementary Figure S9. Whether or Not TL Was Performed: Using TL**

A) Forest plots of sensitivity and specificity for studies employing TL (9 studies, 9 datasets). B) Bivariate boxplot illustrating distribution and heterogeneity. C) Deeks’ funnel plot assessing potential publication bias. D) Fagan’s nomogram depicting post-test probability. E) Clinical application plot of LRP and LRN.

**
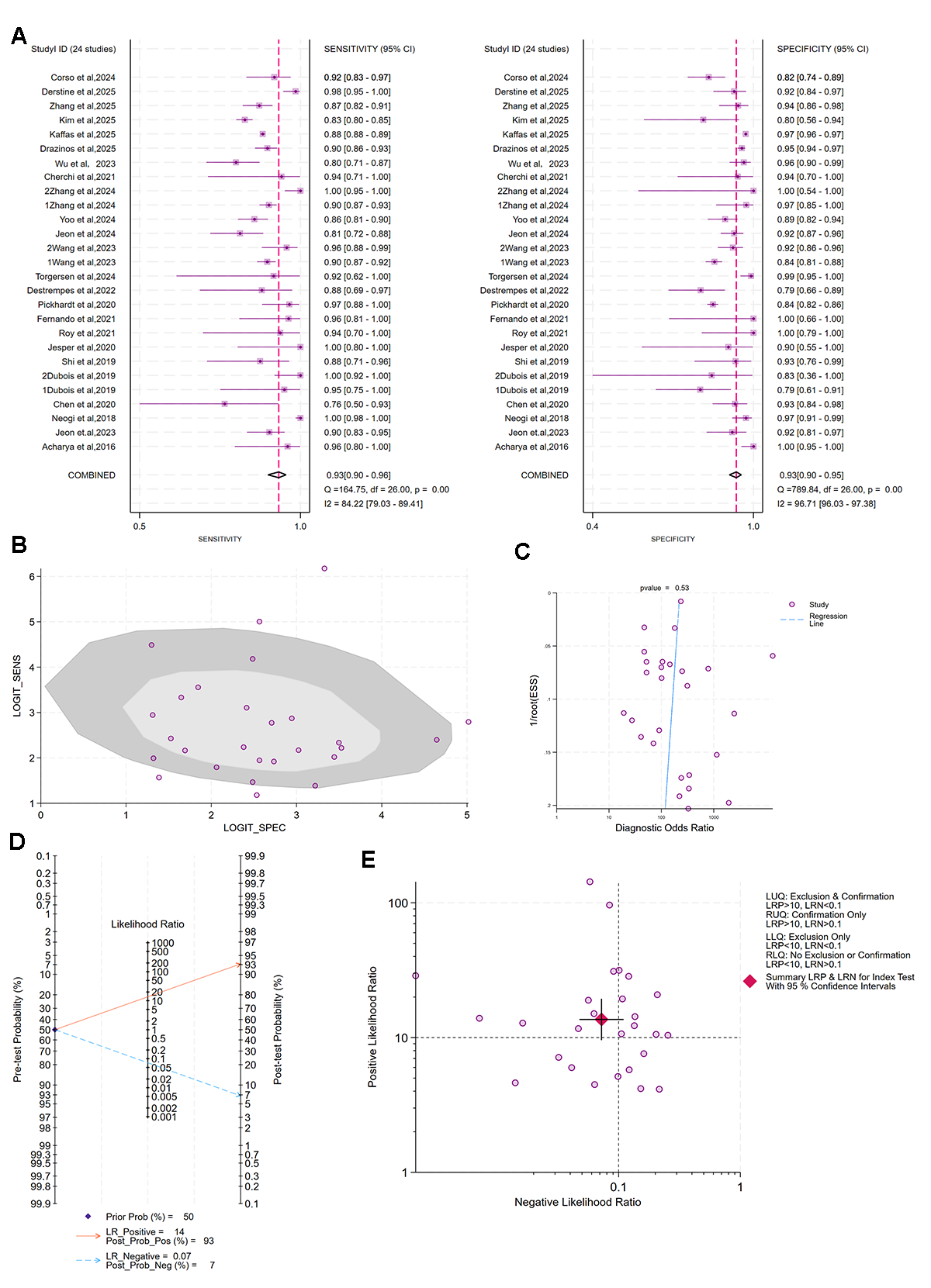
Supplementary Figure S10. Whether or Not TL Was Performed - Not Using TL**

A) Forest plots of sensitivity and specificity for studies not employing TL (24 studies, 26 datasets). B) Bivariate boxplot illustrating distribution and heterogeneity. C) Deeks’ funnel plot assessing potential publication bias. D) Fagan’s nomogram depicting post-test probability. E) Clinical application plot of LRP and LRN.

**
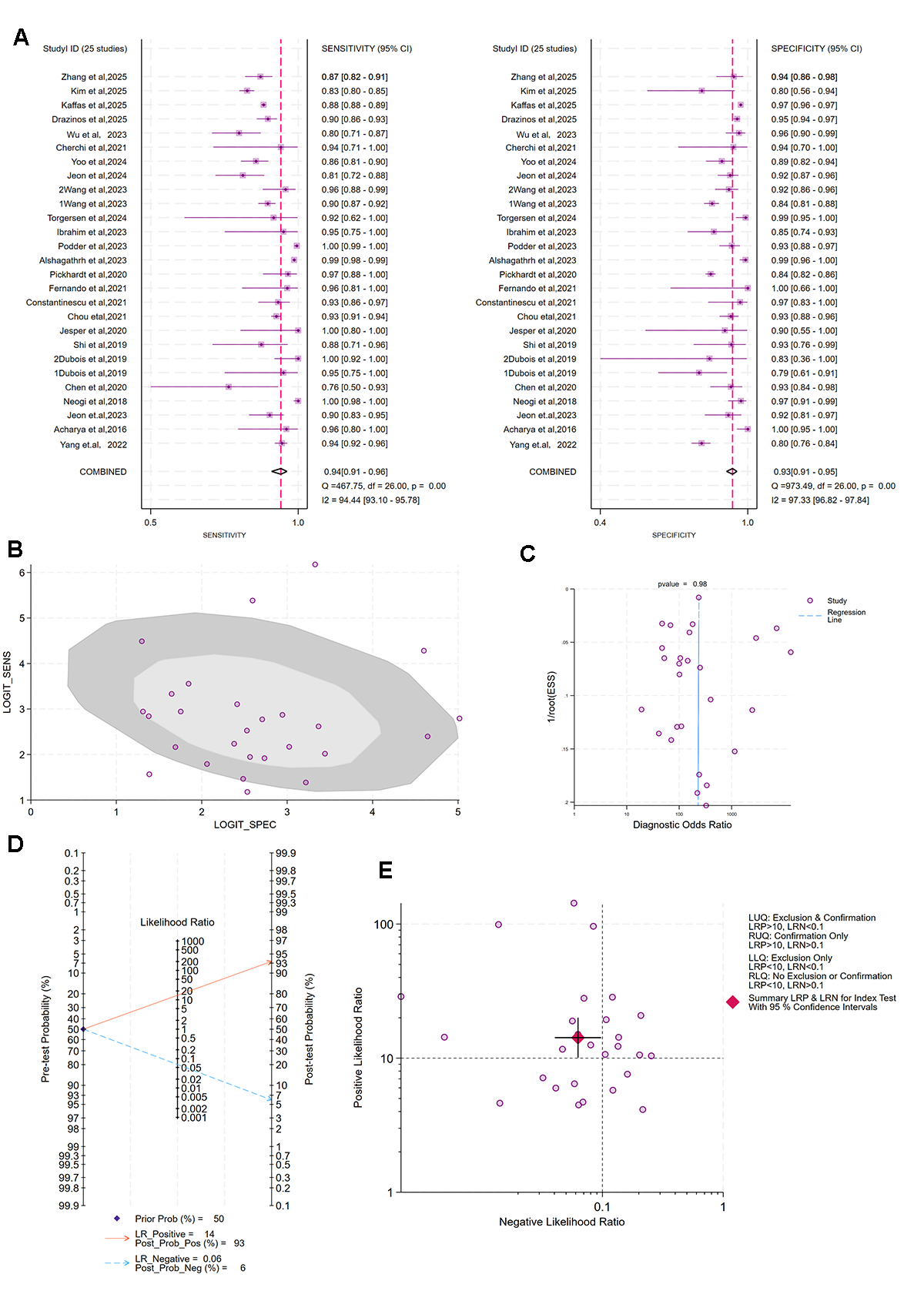
Supplementary Figure S11. Research Design - Single-center**

A) Forest plots of sensitivity and specificity for single-center studies (25 studies, 26 datasets). B) Bivariate boxplot illustrating distribution and heterogeneity. C) Deeks’ funnel plot assessing potential publication bias. D) Fagan’s nomogram depicting post-test probability. E) Clinical application plot of LRP and LRN.

**
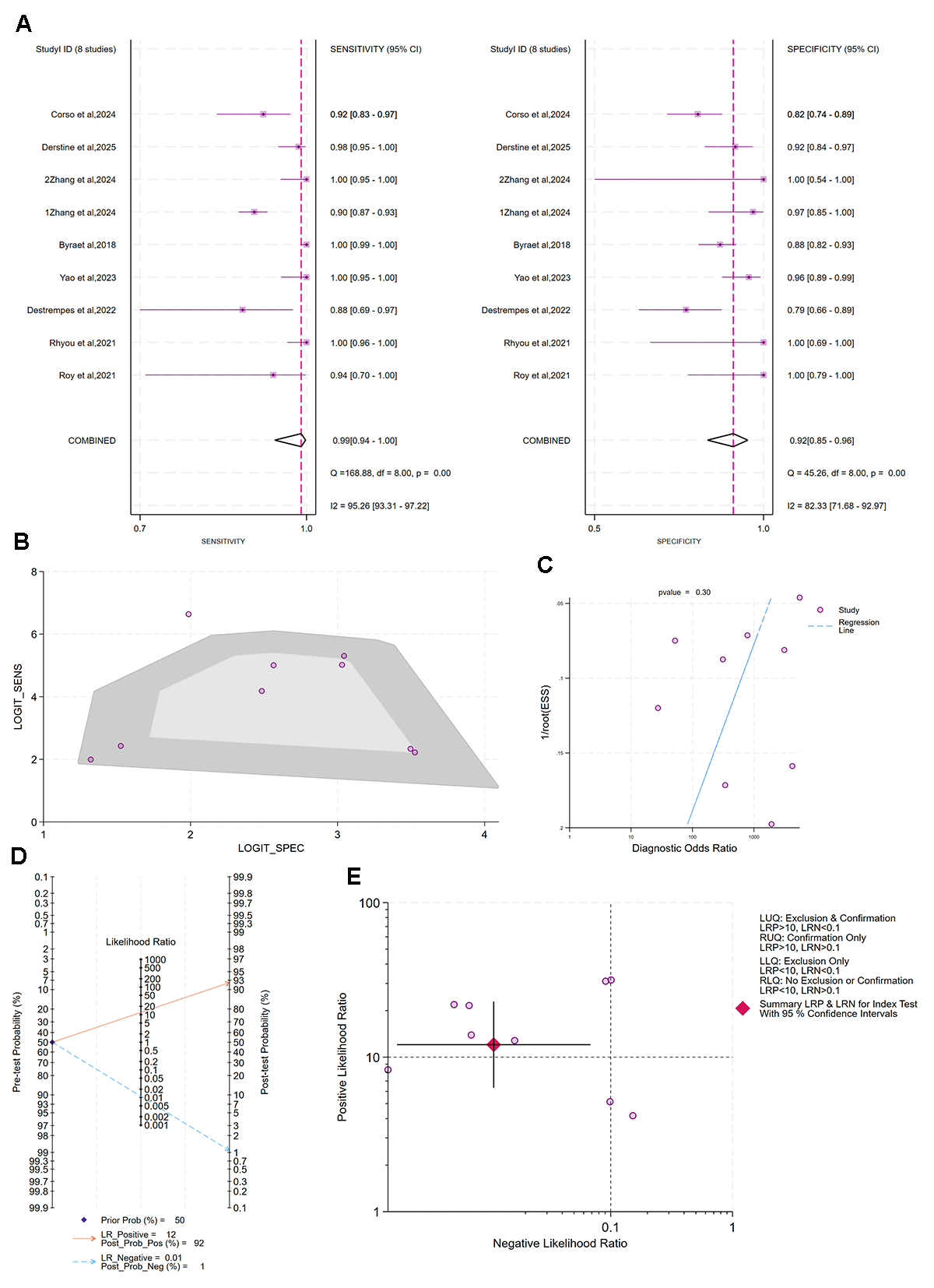
Supplementary Figure S12. Research Design - Multi-center**

A) Forest plots of sensitivity and specificity for multi-center studies (8 studies, 9 datasets). B) Bivariate boxplot illustrating distribution and heterogeneity. C) Deeks’ funnel plot assessing potential publication bias. D) Fagan’s nomogram depicting post-test probability. E) Clinical application plot of LRP and LRN.

**
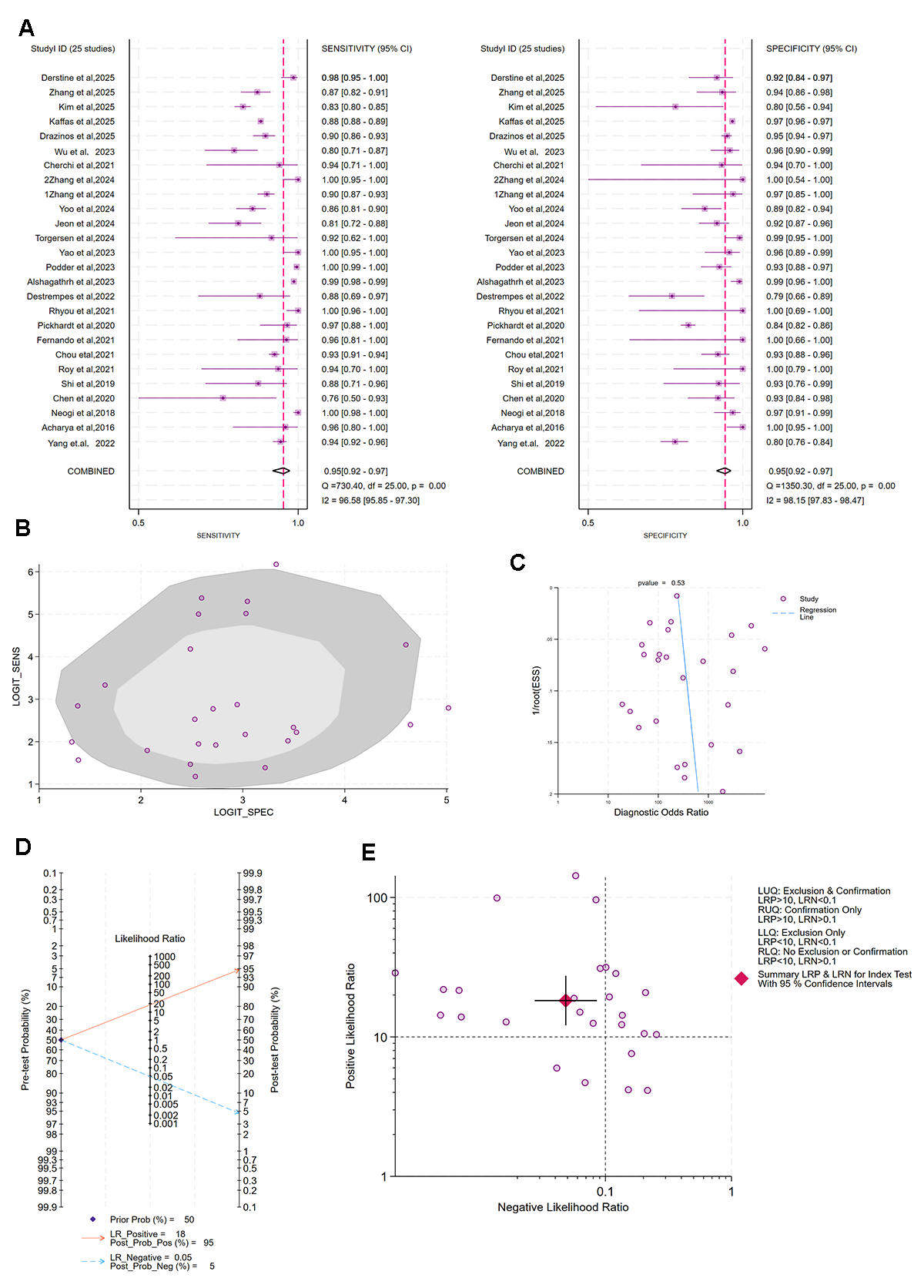
Supplementary Figure S13. Research Type - Retrospective Study**

A) Forest plots of sensitivity and specificity for retrospective studies (25 studies, 26 datasets). B) Bivariate boxplot illustrating distribution and heterogeneity. C) Deeks’ funnel plot assessing potential publication bias. D) Fagan’s nomogram depicting post-test probability. E) Clinical application plot of LRP and LRN.

**
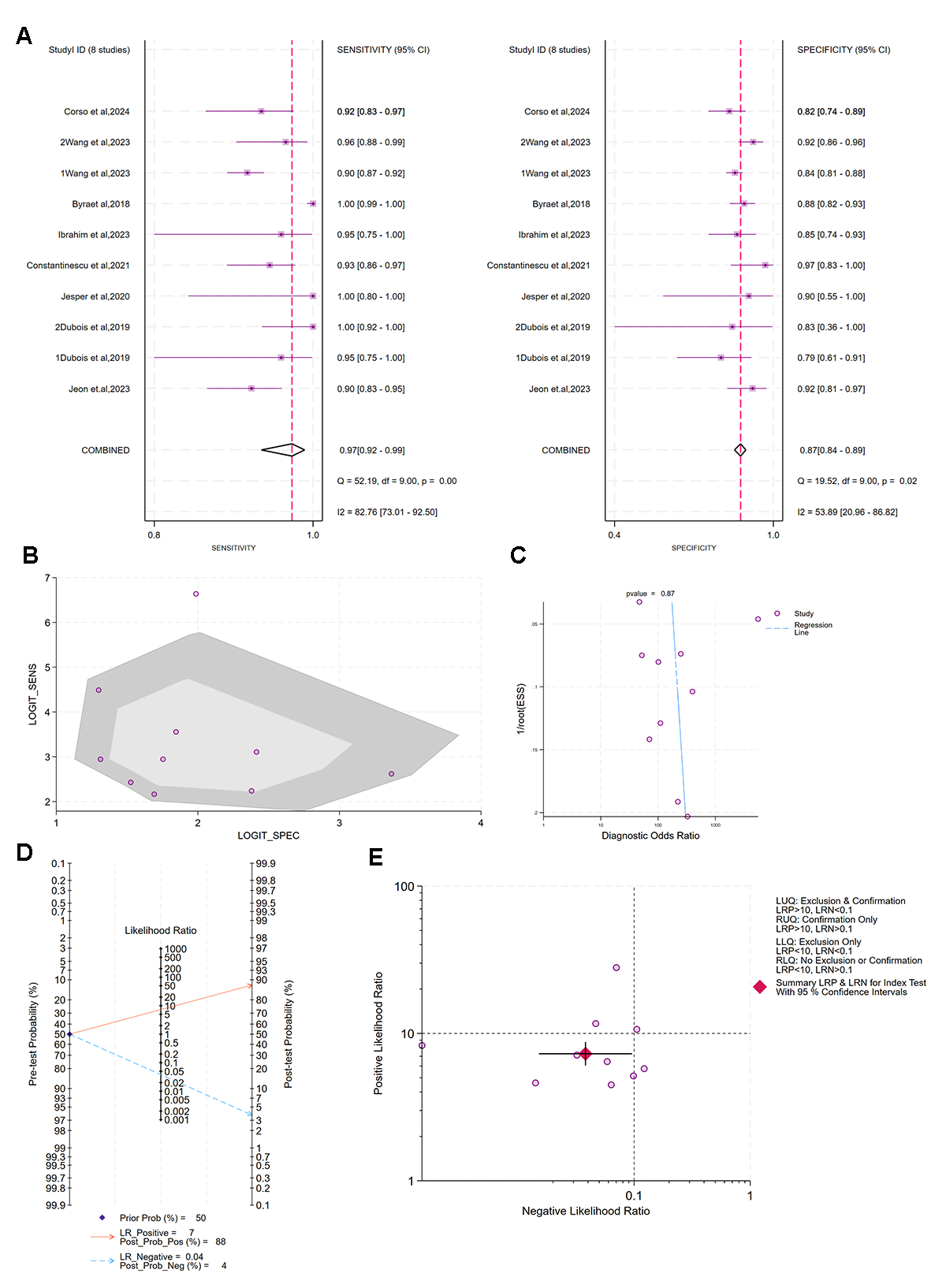
Supplementary Figure S14. Research Type - Prospective Study**

A) Forest plots of sensitivity and specificity for prospective studies (8 studies, 9 datasets). B) Bivariate boxplot illustrating distribution and heterogeneity. C) Deeks’ funnel plot assessing potential publication bias. D) Fagan’s nomogram depicting post-test probability. E) Clinical application plot of LRP and LRN.

**
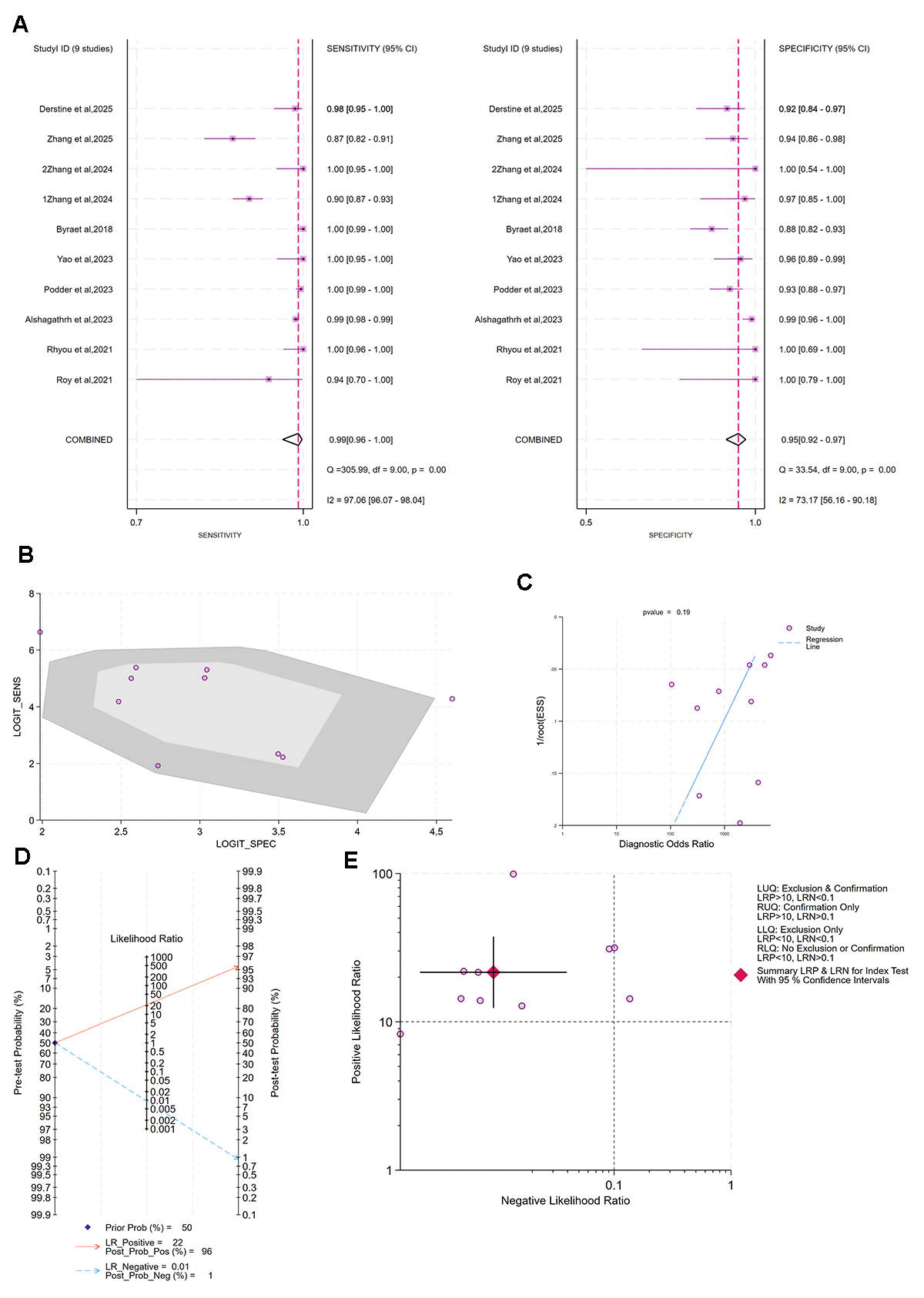
Supplementary Figure S15. Data Availability or Unavailability - Data Availability**

A) Forest plots of sensitivity and specificity for studies with available data (9 studies, 10 datasets). B) Bivariate boxplot illustrating distribution and heterogeneity. C) Deeks’ funnel plot assessing potential publication bias. D) Fagan’s nomogram depicting post-test probability. E) Clinical application plot of LRP and LRN.

**
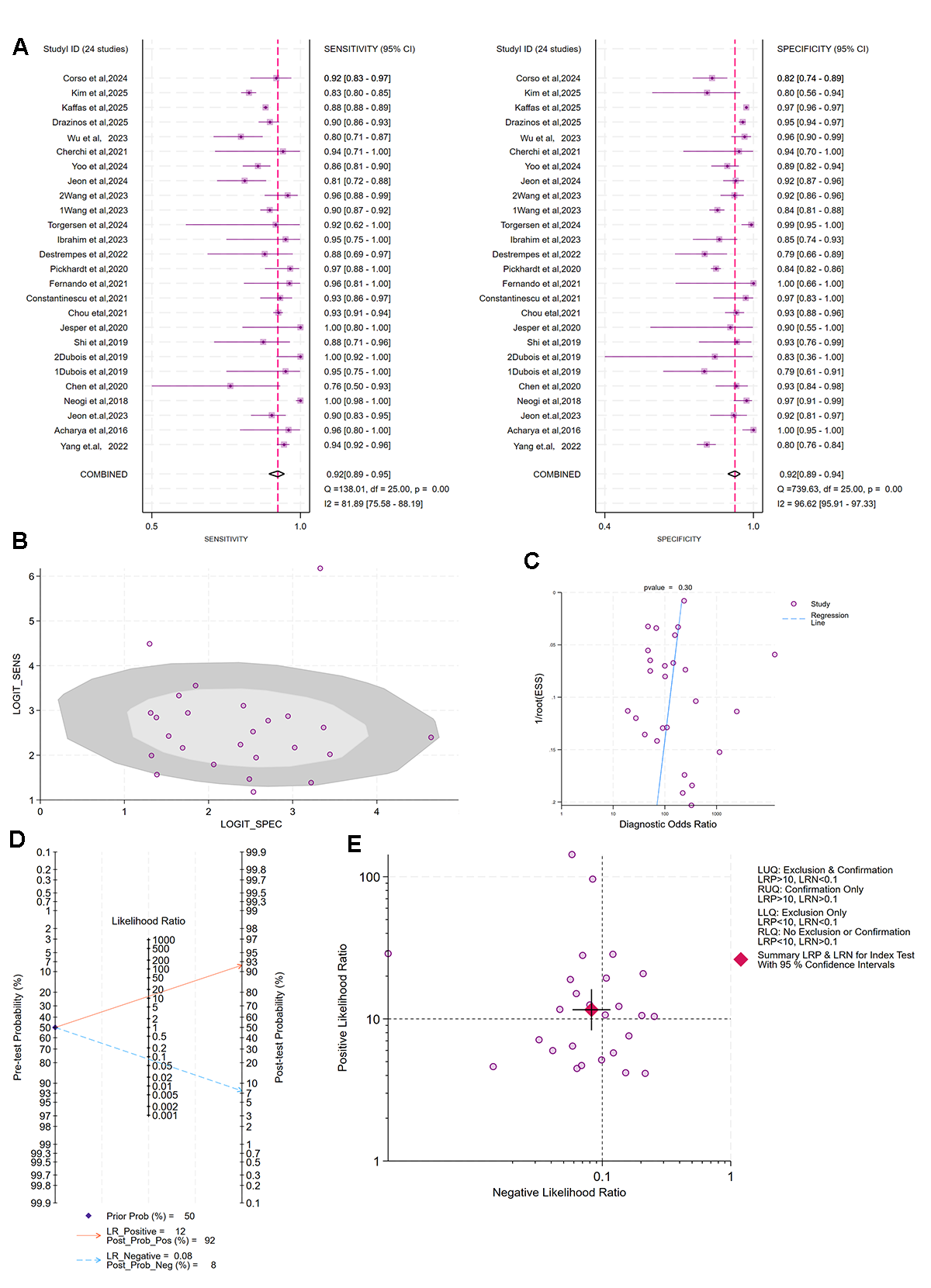
Supplementary Figure S16. Data Availability or Unavailability - Data Unavailability**

A) Forest plots of sensitivity and specificity for studies with unavailable data (24 studies, 25 datasets). B) Bivariate boxplot illustrating distribution and heterogeneity. C) Deeks’ funnel plot assessing potential publication bias. D) Fagan’s nomogram depicting post-test probability. E) Clinical application plot of LRP and LRN.
